# Clinical management of female patients with Fabry disease based on expert consensus

**DOI:** 10.1186/s13023-024-03500-7

**Published:** 2025-01-07

**Authors:** Eva Brand, Aleš Linhart, Patrick Deegan, Ruxandra Jurcut, Antonio Pisani, Roser Torra, Ulla Feldt-Rasmussen

**Affiliations:** 1 Department of Nephrology, Hypertension and Rheumatology, and Interdisciplinary Fabry Centre Münster (IFAZ), University Hospital Münster, Münster, Germany; 2 2nd Department of Internal Cardiovascular Medicine, General University Hospital, Prague, First Faculty of Medicine, Charles University, Prague, Czech Republic; 3 Lysosomal Disorders Unit, Addenbrooke’s Hospital, Cambridge, UK; 4 Expert Center for Rare Genetic Cardiovascular Diseases, Institute of Emergency for Cardiovascular Diseases “Prof. Dr. C.C. Iliescu”, University of Medicine and Pharmacy “Carol Davila”, Bucharest, Romania; 5 Department of Public Health, University Federico II of Napoli, Napoli, Italy; 6 Inherited Kidney Diseases, Nephrology Department, Fundació Puigvert, Institut Recerca Sant Pau (IR-SANT PAU), Universitat Autònoma de Barcelona, Barcelona, Spain; 7 Department of Nephrology and Endocrinology, Rigshospitalet, Copenhagen University Hospital, Copenhagen, Denmark; 8 Institute of Clinical Medicine, Faculty of Health and Clinical Sciences, Copenhagen University, Copenhagen, Denmark; 9 Internal Medicine D, Department of Nephrology, Hypertension and Rheumatology and Interdisciplinary Fabry Centre Münster (IFAZ), University Hospital Münster, Albert-Schweitzer-Campus 1, 48149 Münster, Germany

**Keywords:** Lysosomal storage disease, Inherited metabolic disease, Kidney diseases, Fabry disease, Female, Early diagnosis, Enzyme replacement therapy, Patient-reported outcome measures

## Abstract

Fabry disease is an X-linked lysosomal storage disorder that causes accumulation of glycosphingolipids in body tissues and fluids, leading to progressive organ damage and life-threatening complications. It can affect both males and females and can be classified into classic or later-onset phenotypes. The disease severity in females ranges from asymptomatic to the more severe, classic phenotype. Most females are hemizygous and the X-linked inheritance is associated with variable X-activation pattern and residual enzymatic activity. The heterogeneity of clinical presentation in females requires different approaches to diagnosis and management than males. A European group of 7 physicians, experienced in the management of Fabry disease, convened to discuss patient perspectives and published guidelines. The experts discussed the need to focus on psychological treatment in relation to individual coping styles when monitoring targets, and the lack of data supporting the use of plasma globotriaosylsphingosine over enzyme activity in the diagnosis of these patients. It was suggested that the high phenotypic variability in female patients may be related to the dynamic nature of the X-chromosome inactivation process and further understanding of this process could help predict the progression of Fabry disease in females and facilitate timely intervention. Due to the range of disease severity they exhibit, female patients with Fabry disease may require a more individualized treatment approach than males. Despite current recommendations, the experts agreed that early disease-specific treatment initiation in high-risk females could improve clinical outcome.

**Supplementary Information:**

The online version contains supplementary material available at 10.1186/s13023-024-03500-7.

## Background

Fabry disease (FD; OMIM 301500) is a rare, X-linked lysosomal storage disorder caused by pathogenic variants in the *GLA* gene encoding the enzyme α-galactosidase A (α-Gal A) [1]. Deficient or absent α-Gal A enzyme and the subsequent lysosomal accumulation of glycosphingolipids cause progressive organ damage with life-threatening complications and increased risk of premature death [2]. 

FD can be broadly classified into two phenotypes: “classic” and “non-classic” (including later-onset) [2]. The phenotypic variability of disease manifestations is high in female patients, with symptoms/manifestations usually occurring later in life and exhibiting slower disease progression [3]. Clinical experience of females carrying *GLA* pathogenic variants has shown a range of phenotypes – from an asymptomatic to a severe classical phenotype, with cardiac, renal, and neurologic involvement, as well as impaired quality of life (QoL) [4, 5]. In addition, among female patients with FD bearing the same mutation, different organs can be affected to different degrees.

This variability could be, in part, due to the proportion of random X-chromosome inactivation (XCI) of the X chromosome carrying the allele with pathogenic mutation which may differ between individuals globally or even within a particular organ in the same individual [6, 7]. In the case of FD, there are conflicting results regarding the XCI profiles of heterozygous females [8]. A possible explanation for the apparent discrepancies is offered by emerging evidence on the dynamic nature and complexity of the XCI phenomenon and potential methodological issues in XCI assessments [6, 7]. 

Since 2001, enzyme replacement therapy (ERT) with human α-Gal A has been the mainstay of FD-specific treatment to prevent or slow disease progression and in 2016, in Europe, a chaperone therapy was granted marketing authorization for treatment in patients with amenable *GLA* variants [9, 10]. More recently, the European Medicines Agency (EMA) and the Food and Drug Administration (FDA) have approved a pegylated ERT (pegunigalsidase alfa) for the treatment of adults with confirmed FD [11, 12]. Due to the heterogeneous clinical picture of female patients with FD, including those affected females with near normal or normal plasma α-Gal A activity, the optimal time for treatment initiation remains controversial. Current guidelines and recommendations suggest ERT should be initiated after the onset of the first FD-typical renal, cardiac, and/or cerebral clinical manifestations in female patients with classic mutations [2, 13]. However, published evidence has shown ERT to be most effective when started before the onset of irreversible tissue damage [2]. 

Seven European clinical experts were presented with the results of interviews with female patients with FD conducted by Sanofi (details of which are presented in Supplementary Material). They discussed current guidelines and reasonable response to therapy relevant to female patients with the aim of developing a set of organ-specific management goals for these patients. As the presentation and severity of FD in females is variable, this report aims to provide expert opinion and guidance on the management of females with FD, based on published evidence and insights into the experience of these patients.

### Published guidance on management and monitoring of females with FD

The diagnosis of FD should involve a detailed patient history, family history, physical examination, genetic testing, and identification of clinical and biochemical findings suggestive of FD [14]. The clinical manifestations and symptoms suggestive of FD in female patients are summarized in Table 1. In patients with a genetic variant of unknown significance or in patients where it is difficult to interpret the *GLA* variant (e.g., non-specific clinical manifestations or symptoms), family segregation, manifestations in a male family member, as well as various imaging procedures, renal and/or skin biopsy, and expert consultation, may be useful to confirm or exclude the pathogenic nature of the *GLA* variant [15]. In addition, blood concentrations of globotriaosylsphingosine (lyso-GL3), a deacylated form of the main storage molecule globotriaosylceramide (GL3) and a biologically active and water-soluble marker, may be used to stratify high-risk patients who require intensive monitoring and treatment [16]. Although not all female patients will exhibit a high plasma lyso-GL3 concentration [17, 18], studies by Ouyang et al. and Duro et al. found that plasma lyso-GL3 concentrations can be more useful than enzyme assays in diagnosing female patients with FD [18, 19]. However, it is important to note that the data supporting the use of plasma lyso-GL3 over enzyme activity in the diagnosis of these patients is limited. Additional biomarkers for monitoring cardiac and renal disease in all patients with FD can be found in the recent expert consensus paper by Burlina et al. on the recommendations for the use of biomarkers in FD and appear in Table 1 [20]. It is important to note that although female patients tend to have a milder disease phenotype than men, they can still experience cardiac manifestations but these generally appear later in life compared to men [21]. Furthermore, female patients may develop myocardial fibrosis without the accompanying hypertrophy observed in men, which can complicate diagnosis and monitoring [22]. 

**Table 1:** Clinical findings suggestive of Fabry disease and differential diagnosis for female patients with Fabry disease

Organ system	Signs/symptoms	Clinical work-up findings	Differential diagnoses
Renal [2, 31, 32]	Early stages of renal failure: no symptoms Advanced stages of renal failure: peripheral edema (rare), hypertension Uremic symptoms: nausea, vomiting, loss of appetite, dizziness, fatigue, convulsions, coma	**Urine**: Albuminuria (albumin-creatinine-ratio in spontaneous urine; A1-A3) /proteinuria (protein-creatinine ratio in spontaneous urine) **Blood**: hyperfiltration or decreased glomerular filtration rate determined by eGFR (G1-G5) **Kidney ultrasound**: parapelvic cysts only in men with classic FD	Primary or secondary glomerulonephritis
Cardiac [2]	Heart failure, palpitations, syncope, sudden cardiac death	**Cardiac imaging** (ECHO, CMR): hypertrophic cardiomyopathy; low longitudinal myocardial function; low longitudinal strain (mainly at basal inferolateral wall level); cardiac fibrosis (LGE mainly at basal inferolateral wall level on cardiac MRI); low T1 values at native MRI T1 mapping **ECG**: short PR, LVH, ST-T changes, bradycardia, chronotropic incompetence, cardiac arrhythmia (atrial fibrillation, non-sustained ventricular tachycardia) **Biomarkers**: high BNP/NT-proBNP, high-sensitivity troponin	Sarcomeric hypertrophic cardiomyopathy, amyloidosis, hypertensive cardiac disease
Vascular [2]	Aortic stiffness (in males)	High pulse-wave velocity	Patients with risk factors for atherosclerosis, chronic renal insufficiency
Peripheral nervous system [2]	Acroparesthesia	EMG: sensation neuropathy Sudoscan	Other causes such as diabetes mellitus, vitamin B12 deficiency, autoimmune diseases
Central nervous system [33]	Stroke signs: Sudden numbness or weakness in the face, arm, or leg, especially on one side of the body; sudden confusion, trouble speaking, or difficulty understanding speech; sudden trouble seeing in one or both eyes; sudden trouble walking, dizziness, loss of balance, or lack of coordination	Cerebral imaging: white matter lesions, TIA, ischemic stroke, and (less frequently) hemorrhagic stroke Microbleeds Stroke	Multiple sclerosis Cerebral Autosomal-Dominant Arteriopathy with Subcortical Infarcts and Leukoencephalopathy (CADASIL) and Cerebral Autosomal-Recessive Arteriopathy with Subcortical Infarcts and Leukoencephalopathy (CARASIL)
ENT [2]	Dizziness, hearing loss	Vestibular tests Audiogram	
Dermatological [2]	Angiokeratoma		
Ophthalmological [2]	Cornea verticillata	Slit-lamp examination	Induced by certain medications, e.g., amiodarone, hydroxychloroquine
Gastrointestinal [34]	Nausea, vomiting, diarrhea, and constipation; abdominal pain, and/or bloating	Normal endoscopy; biopsy involving submucosa may show infiltration of neural plexus	Non-specific symptoms may suggest more common disorders, e.g., irritable bowel syndrome or inflammatory bowel disease
Neuropsychological [2]	Common: depression, anxiety, panic attacks, social adaptive function difficulties Rare: cognitive decline and dementia		
Pulmonary [2]	Dyspnea, wheezing, dry cough, sleep-disordered breathing	Pulmonary function test, chest X-ray	Chronic bronchitis, COPD
Lymphatic [2]	Lymphedema in all or part of a limb (also below eyes)		Lipedema
Skeletal [2]	Osteopenia, osteoporosis	DEXA scan of bones for bone mineral density	Other causes of osteopenia and osteoporosis
Other [2]	Mild facial dysmorphism		

BNP, brain natriuretic peptide; CMR, cardiac magnetic resonance; COPD, chronic obstructive pulmonary disease; DEXA, dual-energy X-ray absorptiometry; ECG, electrocardiogram; ECHO, echocardiogram; eGFR, estimated glomerular filtration rate; EMG, electromyography; ENT, ear, nose, and throat; FD, Fabry disease; LGE, late-gadolinium enhancement; LVH, left ventricular hypertrophy; MRI, magnetic resonance imaging; NT-proBNP, N-terminal prohormone of brain natriuretic peptide; TIA, transient ischemic attack

Current guidelines recommend that female patients with FD should be treated when the first clinically relevant Fabry disease-associated manifestations occur. Evidence from studies of patients receiving ERT suggests that early diagnosis and timely initiation of therapy can prevent further disease progression which could otherwise lead to irreversible tissue damage and organ failure [2]. The initiation of treatment depends on a patient’s sex, phenotype, and the manifestation of signs and symptoms characteristic of FD. However, due to the range of disease severity they exhibit, female patients with FD may require a more individualized treatment approach than males (Table 2) [2]. The degree of patient monitoring is influenced more by disease severity than by type of treatment. Patients are classed according to their stage of renal disease based on the Kidney Disease Improving Global Outcomes guideline for the management of glomerular diseases [23]. Patients should therefore be monitored at appropriate intervals to effectively track disease progression, particularly adult patients without FD-related clinical symptoms, and guidance suggests that a switch in therapy may be appropriate in patients who do not achieve their treatment goals [2, 24–26]. 

**Table 2:** Organ-specific management goals for females with Fabry disease based on published guidelines and expert opinion [2, 35, 36]

Organ system and associated risk factors	Goal	Reference values
Cardiac	No **LVH**: prevent LVH development	Wall thickness < 10 mm, LVMi < 95 g/m^2^ (echocardiographic definition)
	LVH present: prevent further progression/achieve LVH stabilization, prevent complications	
	No **fibrosis**: prevent fibrosis development	
	Fibrosis present: prevent further progression/achieve stabilization	
	**Atrial fibrillation**: prevent development by stabilizing LVH/fibrosis; monitor for their presence; treat as soon as detected; anti-coagulate for stroke prevention regardless of calculated thrombotic risk (CHA_2_DS_2_-VASc)	
	**Ventricular arrhythmias**: prevent development by stabilizing LVH/fibrosis; monitor for their presence; treat as soon as detected; prevent SCD by ICD implantation in primary or secondary prevention	
	**Conduction disorders**: prevent development by stabilizing LVH/fibrosis; monitor for their presence; treat high-degree AV block with cardiac pacing	
	**Optimal control of conventional CV risk factors**: reduce CV morbidity and mortality by optimal blood pressure control as described in the current guidelines for management of hypertension and modification of other CV risk factors	18–<79 years: 120–130/70–80 mmHg
		≥ 80 years: 130–140/70–80 mmHg
Renal	**No or mild kidney involvement (eGFR > 90 ml/min/1.73 m**^**2**^**)**: eGFR/mGFR should be maintained in an age-appropriate normal range	
	**Mild-to-moderate (eGFR 60–90 ml/min/1.73 m**^**2**^**), moderate-to-severe (eGFR 30–60 ml/min/1.73 m**^**2**^**) and severe (eGFR 15–30 ml/min/1.73 m**^**2**^**) kidney function impairment**: prevent progression of eGFR loss and stabilize GFR levels/avoid the need for kidney-replacement therapy	Depending on CKD stage and progression: Stabilization: GFR loss ≤ 1–3 ml/min/1.73m^2^; slow the process of progression (> 3 ml/min/1.73 m^2^) or fast progression (> 5 ml/min/1.73 m^2^) to an annual decrease of < 3 and < 5 ml/min/1.73 m^2^, respectively
	In case of rapid kidney function deterioration, verify the cause by kidney biopsy in the event that this is non-Fabry related **ESKD**: provide optimal kidney-replacement therapy and avoid cardiac and cerebral damage	
	Maintain ERT to avoid cardiac and cerebral damage while on kidney-replacement therapy	
	Suggest kidney transplantation before dialysis	
	Keep **albuminuria** levels as low as possible	Normal albuminuria levels: <30 mg/g
	Use adjunctive therapies (e.g., ACEi or ARB, finerenone and SGLT2 inhibitors) in addition to ERT to normalize/stabilize kidney function with albuminuria (reduce levels to < 300 mg/g)	Microalbuminuria levels: 30–300 mg/g
		Macroalbuminuria levels: >300 mg/g
Neuropathic pain	Optimize pain management	Improvement in Brief Pain Inventory score
	Attempt to avoid lesions, and be aware of and manage pain due to nerve compression/entrapment (e.g., carpal tunnel syndrome)	
Gastrointestinal dysfunction	Avoid or reduce GI symptoms and prevent progression of symptoms	
	Monitor GI symptoms using validated GI rating scales (Rome III, Gastrointestinal Symptom Rating Scale, Fabry disease Patient-Reported Outcome-Gastrointestinal or the Bristol Stool Form Scale)	
Ophthalmological manifestations	Manage dry-eye symptoms in patients with symptomatic conjunctival lymphangiectasia with usual treatments	

ACEi, angiotensin-converting enzyme inhibitor; ARB, angiotensin receptor blocker; AV, atrioventricular; CHA_2_DS_2_-VASc, congestive heart failure, hypertension, age, diabetes mellitus, prior stroke/transient ischemic attack, vascular disease, age, sex score; CKD, chronic kidney disease; CV, cardiovascular; eGFR, estimated glomerular filtration rate; ERT, enzyme replacement therapy; ESKD, end-stage kidney disease; GFR, glomerular filtration rate; GI gastrointestinal; ICD, implantable cardioverter-defibrillator; LVH, left ventricular hypertrophy; LVMi, left ventricular mass index; mGFR, measured glomerular filtration rate; mmHg, millimeters of mercury; SCD, sudden cardiac death; SGLT2, sodium-glucose cotransporter-2

### Expert opinion on treatment and management goals: results from the clinical experts’ advisory board

The advisors discussed the guidelines and their experience in diagnosing, treating, and monitoring female patients with FD. All advisors agreed that genetic testing to identify *GLA* gene variants and blood tests measuring activity of α-Gal A enzyme and lyso-GL3 concentrations are key examinations used to aid the diagnosis of FD in all patients. Most clinical experts (n = 5/7) agreed that lyso-GL3 could be a relevant prognostic marker for assessing disease severity and progression; however, it is currently only validated for use in diagnosis. The experts noted that the decrease in lyso-GL3 after treatment initiation is typically steeper in males than in females, which may be explained by males having a higher baseline lyso-GL3 value.

For female patients with FD, cardiac disease was the feature most mentioned, although it was noted that disease features can vary between individuals depending on age and type of variant. Additionally, the experts agreed that if any patient presents with low estimated glomerular filtration rate (eGFR) without albuminuria/proteinuria, the underlying cause is less likely to be FD and requires detailed investigation (Table 3). Early organ involvement and treatment-refractory neuropathic pain were the most frequently mentioned reasons for initiating treatment in female patients with FD. The clinical experts concluded that the extent of monitoring depends on the degree of organ involvement; stable patients on treatment are monitored annually, symptomatic patients with disease progression twice per year, and untreated, asymptomatic, stable female patients every other year (Table 3). Encouragingly, most experts within this group believe that female patients with FD are being treated according to current guidelines.

**Table 3:** Recommended monitoring schedule in female patients with Fabry disease based on guidelines and expert opinion [2, 24, 25]

Organ system	Examination	Monitoring schedule
General	Physical examination	
	Evaluation of QoL (SF-36 or EQ5D)	Every clinic visit, at baseline and at shift of treatment
	School/work performance, level of depression and anxiety	
	Severity Score Index for adults – MSSI or FASTEX	
General lab variables	α-Gal A enzyme activity and *GLA* variant analysis	At baseline, if not previously determined
	Lyso-GL3	Lyso-GL3 also at the start of therapy and during the course (at least annually)
Cardiac	Blood pressure and cardiac rhythm	Every clinic visit
	ECG	Annually, and as needed
	Holter monitoring	Annually
	Implantable loop recorder	For detailed rhythm surveillance, as needed
	MRI with gadolinium	At diagnosis, at an interval of > 2 years or in case of clinical progression
	BNP/NT-proBNP	Annually for patients with cardiomyopathy or bradycardia
	hs-cTn	As a surrogate for myocardial fibrosis
	Coronary angiography – CT or invasive	If patient has clinical signs of angina pectoris
Renal	eGFR (using appropriate formulae)	Annually if low risk, every 6 months if moderate risk, and every 3 months if high to very high risk*
	Albuminuria and/or proteinuria, sodium, potassium, and creatinine	
	Renal biopsy	As clinically indicated, e.g., in case of unexplained eGFR decline or progressive microalbuminuria/proteinuria and for evaluation of possible secondary renal diseases (comorbidities)
Cerebrovascular	Brain MRI	Every 3 years and when clinically indicated
	PET-CT or CT imaging	In case of acute stroke and if MRI is contraindicated due to cardiac pacing
	Comorbid stroke risk factors: cholesterol (total, LDL, HDL), triglycerides, lipoprotein A, total plasma homocysteine, factor V Leiden (G1691A), Protein C and S, prothrombin G20210A, antithrombin III, anticardiolipin antibody, lupus anticoagulant	Baseline for all risk factors and then annually for cholesterol (total, LDL, HDL) and triglycerides
Autonomic nervous system	Tilt test, beat-to-beat variability and sweat test	As required
Gastrointestinal system	Postprandial abdominal pain, bloating, diarrhea, nausea, vomiting, early satiety, difficulty gaining weight	Annually (depending on the complaints refer for endoscopic evaluation if needed)
Peripheral nervous system	Pain evaluation, cold and heat intolerance, vibratory thresholds	Annually
ENT	Audiometry, tympanometry, otoacoustic emissions	As required
	Tinnitus, vertigo, dizziness	Annually
Pulmonary system	Cough, exertional dyspnea, wheezing, and exercise intolerance	
	Spirometry, including response to bronchodilators, treadmill exercise, oximetry, and chest X-ray	Baseline and then as required
Ophthalmological manifestations	Ophthalmologic exam (slit-lamp, direct ophthalmoscopy, best corrected visual acuity, and visual fields)	Annually
	Rational examination	
Skeletal	Bone DEXA scan	Baseline and then as required (particularly in postmenopausal women)

α-Gal A, alpha-galactosidase A; BNP, brain natriuretic peptide; CKD, chronic kidney disease; CT, computed tomography; DEXA, dual-energy-X-ray absorptiometry; ECG, electrocardiogram; eGFR, estimated glomerular filtration rate; ENT, ear, nose, and throat; EQ-5D, EuroQoL 5 Dimensions; FASTEX, FAbry STabilization index; GFR, glomerular filtration rate; GL3, globotriaosylsphingosine; HDL, high-density lipoprotein; hs-cTn, high-sensitivity cardiac troponin; KDIGO, Kidney Disease Improving Global Outcomes; LDL, low-density lipoprotein; lyso-GL3, globotriaosylsphingosine; MRI, magnetic resonance imaging; MSSI, Mainz Severity Score Index; NT-proBNP, N-terminal prohormone of brain natriuretic peptide; PET, positron emission tomography; QoL, quality of life; SF-36, The Short Form 36-Item Health Survey

* Risk levels based on KDIGO 2021 CKD classification scheme. Low risk (CKD stage G1/2 A1), moderate risk (CKD stage G1/2 A2, G3a A1), high (CKD stage G1/2 A3, G3a A2, G3b A1) to very high risk (CKD Stage G3a A3, G3b A2-A3, G4/5 A1-3) [23]

### Future perspectives

#### Importance of genetic counseling and psychological support

Psychological symptoms such as depression are highly prevalent in patients with FD. A study by Ali et al. reported a beneficial outcome from psychological treatment, with improvements in mental health and QoL in eight female patients with FD [27]. Based on this evidence and the interview responses of patients with FD, the experts agreed there is a clinical need for monitoring goals to emphasize psychological treatment tailored to individual coping styles.

#### XCI may help predict the progression of FD in females and facilitate timely intervention

Phenotypic variability of disease is high in female patients. This could be, in part, due to tissue-specific differences in XCI in the same individual, which may be related to cell-specific XCI [6, 7]. In the case of FD, there are conflicting results regarding the XCI profiles of heterozygous females, resulting in variable *GLA* gene expression [8]. A possible explanation of such apparent discrepancies is offered by emerging evidence on the dynamic nature and complexity of the XCI process, which may have more impact than previously believed [6]. This complexity includes a proportion of genes escaping XCI, tissue-specific and cell-to-cell differences of the XCI process, and the intricate gene-specific and gene region-specific role of DNA methylation, which influences gene expression, exerted on both active X (Xa) and inactive X (Xi) chromosomes [6]. A study conducted by Echevarria et al. found that patients with skewed XCI ratios and predominant expression of the pathogenic *GLA* allele had very low or absent residual enzyme activity [8]. This study also revealed a significant and calculable correlation between the XCI direction observed in the blood with that present in other tissues studied (skin, buccal smears, urinary epithelia). Significant differences in residual α-Gal A, disease severity scores, and cardiac and renal disease, dependent on the direction and degree of skewing, were also identified, implying that XCI could significantly impact the phenotype and natural history of FD in female patients [8]. 

The existence of skewed XCI in females with FD was demonstrated by analyzing the human androgen receptor (*HUMARA*) gene; this revealed the existence of skewed inactivation in 16 heterozygous females correlating with FD severity scores [8, 28]. 

A study conducted by Hossain et al. demonstrated a correlation between the methylation state of the wild-type *GLA* allele and the early onset and severity of disease manifestations in a heterozygous female with acroparesthesia, facial dysmorphism, left ventricular hypertrophy, and intellectual disability, in addition to a proven family history particularly relevant to FD [29]. Another study found a correlation between disease severity, lyso-GL3 accumulation, and methylation of the normal allele [28]. 

Most studies investigating the role of DNA methylation in FD defined the extent and direction of XCI through *HUMARA* testing as it is inexpensive and fast [6]. However, it is important to note that *HUMARA* testing cannot distinguish between the two chromosomes and thus it cannot predict disease outcome in a female patient with very skewed XCI. In addition, allele-specific DNA methylation at the promoter region of the *GLA* gene may also influence expression levels of the mutated allele, impacting the onset and progression of FD [6]. Therefore, approaches that distinguish between the mutated and non-mutated allele when analyzing DNA methylation at the *GLA* promoter may be much more informative [6]. Řeboun et al. studied XCI in 35 female patients with FD using two methylation-based and two allele-specific expression assays in combination with *GLA* expression analyses, and enzyme activity [7]. This study showed a good concordance of methylation and allele-specific expression assays for assessment of XCI in females with FD. Furthermore, Řeboun et al. found correlating XCI to *GLA* expression and α-Gal A activity facilitated identification of crossing-over between the loci used for methylation assays and the *GLA* locus. Combining XCI assays, *GLA* expression analyses, and enzyme activity may potentially minimize technical and biological pitfalls [7]. 

The hope for the future is that *ad hoc* and ultra-deep methylation analyses of the *GLA* gene will provide epigenetic signatures that will help predict the disease course for females with FD, thus allowing timely interventions.

## Conclusions

In conclusion, the clinical heterogeneity of FD requires an individualized approach to patient care that reflects the genotype, sex, age, family history, phenotype, and specific clinical disease severity of each patient. The management of female patients with FD should involve timely disease-specific treatment (ERT or chaperone therapy, as appropriate), regular assessment of disease progression in all patients, and the use of a multidisciplinary care team to assist in the management of organ-specific complications. Currently, ERT is the cornerstone of therapy, however, randomized trials demonstrate that migalastat may also be prescribed for female patients with amenable mutations although, the response may not be uniform depending on residual enzymatic activity and degree of amenability [30]. Despite growing evidence that the clinical response to treatment in high-risk females is improved with early treatment initiation, current guidelines suggest treatment in females should be initiated after the first signs of cardiac and/or renal disease, central nervous system involvement, gastrointestinal symptoms, and/or rapidly progressive disease. Future observational studies should aim to evaluate the prognostic value of elevated plasma lyso-GL3 for ERT initiation, as a way to avoid delay in treatment initiation is considered an unmet need. In addition, psychological support during the diagnosis, management, and monitoring of FD should be offered to all patients.

In future, *ad hoc* and ultra-deep methylation analyses of the *GLA* gene should provide epigenetic signatures predictive of whether female heterozygotes will develop disease-specific symptoms, identifying opportunities for timely therapeutic intervention. Further education and increased awareness of the signs and symptoms, diagnosis, management, and monitoring of FD is needed among healthcare professionals and the public so that female patients with FD can be better supported.

## Electronic Supplementary Material

Below is the link to the electronic supplementary material.

Additional file 1: Interviews with female patients with Fabry Disease. Methods, discussion guide and qualitative outputs of interviews with female patients with Fabry disease

## Data Availability

Not applicable.

## Abbreviations

α-Gal A: α-galactosidase A
ACEi: Angiotensin-converting enzyme inhibitor
ARB: Angiotensin receptor blocker
AV: Atrioventricular
BNP: Brain natriuretic peptide
CKD: Chronic kidney disease
CMR: Cardiac magnetic resonance
COPD: Chronic obstructive pulmonary disease
CT: Computed tomography
CV: Cardiovascular
DEXA: Dual-energy X-ray absorptiometry
ECG: Electrocardiogram
ECHO: Echocardiogram
eGFR: Estimated glomerular filtration rate
EMA: European Medicines Agency
EMG: Electromyography
ENT: Ear, nose, and throat
EQ5D: EuroQoL 5 Dimensions
ERT: Enzyme replacement therapy
ESKD: End-stage kidney disease
FASTEX: FAbry STabilization index
FD: Fabry disease
FDA: Food and Drug Administration
GI: Gastrointestinal
GL3: Globotriaosylceramide
HDL: High-density lipoprotein
Hs-cTnT: High-sensitivity cardiac troponin T
HUMARA: Human androgen receptor
ICD: Implantable cardioverter-defibrillator
KDIGO: Kidney Disease Improving Global Outcomes
LDL: Low-density lipoprotein
LGE: Late-gadolinium enhancement
LVH: Left ventricular hypertrophy
Lyso-GL3: Globotriaosylsphingosine
MRI: Magnetic resonance imaging
MSSI: Mainz Severity Score Index
NT: N-terminal
PET: Positron emission tomography
QoL: Quality of life
SCD: Sudden cardiac death
SF-36: The Short Form 36-Item Health Survey
TIA: Transient ischemic attack
Xa: Active X
Xi: Inactive X
XCI: X-chromosome inactivation
