# Supplementary material for: Clinical management of female patients with Fabry disease based on expert consensus
Source: Orphanet J Rare Dis. 2025 Jan 7;20:7. doi: 10.1186/s13023-024-03500-7 (PMC11707893; doi:10.1186/s13023-024-03500-7)
Supplement: Supplementary file 1 — Additional file 1: Interviews with female patients with Fabry Disease. Methods, discussion guide and qualitative outputs of interviews with female patients with Fabry disease [file 13023_2024_3500_MOESM1_ESM.docx]

# Appendix: Supplementary material

# Interviews with female patients with Fabry disease (FD)

## Methods

Twelve 45-minute telephone interviews were conducted between December 2020 and January 2021 with patients aged between 29 and 69 years in their native language (United Kingdom, n=7; Germany, n=5). A discussion guide was developed, aiming to explore each patient’s experience of the diagnosis, treatment, and management of FD. The interviews were conducted under General Data Protection Regulation, British Healthcare Business Intelligence Association and European Pharmaceutical Market Research Association guidelines.

## Discussion guide for interview with females with FD

| SECTION 1: Background *(2 minutes)* |
| --- |

*Section objective: To confirm key background details and to ease the respondent comfortably into the discussion.*

1. To begin, I’d like you to tell me a little bit about yourself. Please can you introduce yourself? (Prompt)
   1. How long have you known that you have Fabry disease?
   2. Do you know if anyone else in your family has the condition?

| SECTION 2: Symptom presentation *(up to 10 minutes)* |
| --- |

*Session objective: To understand the patient’s symptom onset and burden prior to diagnosis, and input from healthcare providers (HCPs) and or other groups.*

1. Had you been experiencing any symptoms before you were diagnosed with Fabry disease? *(Prompt)*
   1. How did you feel?
   2. How did these symptoms affect your daily life?
   3. At what point did the symptoms cause you to seek help?

***If patient reports no symptoms prior to diagnosis, e.g., their condition was asymptomatic and Fabry disease was diagnosed as a result of familial screening (including genetic testing), go to Q6.***

1. Which healthcare provider (HCP) did you see initially? *(Prompt)*
   1. Did you see them more than once?
   2. How long did you see them for?
   3. Do you feel like you were given enough help and support at that early stage?
2. How challenging did you find it to be experiencing symptoms without knowing why? What information did you feel you needed at this stage? *(Prompt)*
   1. Did your HCP give you that information?
   2. Did you have to, or choose to, look for that information elsewhere?
   3. Where did you look?
3. Did you take any action as a result of any information you were given or found? *(Prompt)*
   1. What did you do – speak to patient groups, join online forums, or something different?
   2. How did all this information make you feel?
   3. Were you referred by your initial HCP to hospital or a specialist as a result of what you learnt?

| SECTION 3: Diagnosis *(15 minutes)* |
| --- |

*Session objective: To understand the diagnostic journey experienced by the patient, awareness of Fabry disease before and after the definitive diagnosis, and the psychological/emotional impact of being informed of their condition*

Thank you for telling me about what life was like before your diagnosis. I’d really appreciate it if we could now talk about what happened in the lead-up to you being diagnosed.

1. Which HCP did you see – was one or more than one specialist involved? *(Prompt)*
   1. Do you remember what sort of tests you had?
   2. How many consultations did you have from your first visit to the point where Fabry disease was first suspected?
   3. How many consultations did you have from when Fabry disease was first suspected to the point of final diagnosis?
   4. How long a time period was it from first symptoms to diagnosis?
   5. Would you mind telling me how old you were?
2. Which specialist made the diagnosis? *(Prompt)*
   1. How did they explain it to you?
   2. Did they explain about your phenotype?
   3. How did you feel when you were told that you had Fabry disease compared with how you felt before you knew?
   4. Were you happy with the support the specialist gave you through all the investigations and when they told you about your condition?
3. How did your family and friends feel when you told them you had Fabry disease? *(Prompt)*
   1. Did anyone in your family have Fabry disease already? And was understanding your family tree part of the diagnosis?
   2. Was anyone in your family offered genetic screening as a result of your diagnosis?
   3. How did they feel about the diagnosis?
   4. What were the challenges you faced in telling them?
   5. How did you approach discussing this with your children?
   6. When considering your family situation, how impactful was it to discover you had an inherited condition? Did this come up in the discussion with your existing family?
   7. When considering you have an inherited condition, how did you feel about planning a family of your own?
4. Had you known anything about Fabry disease before you were diagnosed? *(Prompt)*
   1. When you were eventually diagnosed, did the HCP give you any information or did you find out more in other ways?
   2. Did you eventually feel like you had enough information to understand and manage your condition?
   3. Did you have the right information to provide to friends and family?
5. Once you’d learnt more about Fabry disease from your HCP, what happened next? *(Prompt)*
   1. Were you referred for any extra support, like patient support groups, societies, genetic counseling, or psychological support? What was the nature of this support?
   2. Did you join any online forums or attend any specialist meetings?
   3. How helpful was the support you did get? Did it change the way you felt about your diagnosis?
   4. Do you feel that there could have been more support or help?
   5. What could pharma companies do to provide some of this support?

| SECTION 4: Treatment *(5 minutes)* |
| --- |

*Session objective: To learn about Fabry-specific and supportive treatments the patient may have received following their diagnosis.*

Thank you for telling me about how you were diagnosed with Fabry disease. I’d like us to move forward now and talk a little about any treatment you had after your diagnosis.

1. Did you start any medical treatment after you had been diagnosed? *(Prompt)*
   1. How long after the diagnosis did this/these start?
   2. What treatment were you given?
   3. How involved were you in the decision-making regarding your treatment?
2. If your treatment is an infusion, would you be open to having your infusion done at home or open to self-infusion?
   1. Would you have any concerns with home/self-infusion?
   2. What would be the advantages for you if you had home or self-infusion?
3. Did you feel supported by the HCP prescribing this treatment and looking after you? *(Prompt)*
   1. Were you given enough information about your treatment(s)?
4. What sort of impact did treatment have on your daily life when you first started? *(Prompt)*
   1. Were there any challenges when treatment first began and are you experiencing any now?
   2. Looking back, how do you think treatment has affected you overall?

| SECTION 5: Long-term management *(10 minutes)* |
| --- |

*Session objective: To obtain deeper insights into the experiences, challenges, and unmet needs of patients with Fabry disease over the long term.*

Thank you for sharing how treatment has been for you. In this last part of the interview, I’d like to explore how things have been for you overall since you were diagnosed with Fabry disease and what changes you’d like to see for all patients with this condition, based on your own experiences.

1. What has been the biggest challenge when it comes to managing your condition? *(Prompt)*
   1. How do you think your condition has affected aspects of your daily life?
      1. Work
      2. Social life
      3. Family life
      4. Your relationships
   2. Did you have to make any lifestyle changes to help you manage your condition?
2. Do you feel like you have enough information and that you’re in control of your condition now? *(Prompt)*
   1. How happy are you with your long-term management by your HCP and follow-ups? What could be done to improve this?
   2. Do you feel like you have enough information and support if you experience flare-ups or a relapse?
   3. Are you aware of other treatment options or new (technological) innovations you may be able to try if you do flare up or relapse?
   4. With everything that you know about Fabry disease now, what would you tell yourself if you could go back in time to when you first began to experience symptoms, or when the tests and investigations began?
3. Based on your own experiences of having Fabry disease for X years, what do you think is/are the most severely unmet need or needs of patients with Fabry disease?

| SECTION 6: Thank you and close *(2 minutes)* |
| --- |

That concludes our interview. We have covered a lot of really valuable and helpful information here, so thank you for giving up your time to take part. It really is appreciated. Do you have anything to add about what we’ve talked about today?

Thank you very much for your time. It was a pleasure speaking with you.

## Outputs of patient interviews

### Experiences of diagnosis

Most of the female patients who participated in the interviews experienced symptoms such as pain in the hands and feet, fatigue, and gastrointestinal problems as early as childhood (n=10/12). Several patients reported that symptoms occurred before 12 years of age (n=6/10). Some patients sought help during childhood for their symptoms (n=4/9); however, some patients were still diagnosed later in life, suggesting a delay in diagnosis. Many different specialists were involved in confirming an FD diagnosis in this cohort, such as genetic counselors, neurologists, ophthalmologists, a specialist Fabry community nurse, an endocrinologist, cardiologists, and nephrologists. Patients’ families were offered genetic screening (n=11/12); one female patient was informed that her daughter would not be tested until she was 16 years of age. Almost all the female patients mentioned that psychological support and genetic counseling would be/is beneficial following diagnosis (n=11/12). Interestingly, most patients felt that FD prevented them from working or socializing with friends due to the nature of their symptoms (n=7/10).

### Experiences of treatment

All patients were receiving FD-specific treatment, including enzyme replacement therapy (ERT [n=8]) or chaperone therapy (n=4), including one patient who switched from ERT; one patient reported taking pills but did not provide further details. The time from diagnosis to initiation of FD-specific treatment varied greatly among respondents, from immediate prescription (n=5) to up to 12 years, as some patients were offered treatment only when manifestations typical of FD arose. The parents of one patient, who was 12 years of age at the time of diagnosis, felt that infusion would be too burdensome at that stage and treatment was not initiated for 3–4 years. Of the female patients with FD receiving ERT (n=8/12), only one patient preferred to receive infusions in the clinic, whereas other patients (n=6/7) preferred home infusion.

Just under half (n=4/10) of the patients experienced a positive impact of their treatment on their daily life, including a reduction in fatigue, pain, and improvements in energy levels, thus improving quality of life (QoL). However, the female patients affected by FD also highlighted that minimizing treatment burden would be likely to have a positive impact on their QoL.

## Summary of patient interviews

Results from the patient interviews highlight the unmet needs faced by female patients with FD. Despite experiencing symptoms during childhood and frequent visits to their general practitioner, many patients were diagnosed with FD at a much later age, highlighting delays in diagnosis. In addition, two patients were only offered treatment after their symptoms/manifestations worsened, reflecting the result of a study by Lenders *et al.*, which found that 33% of females in Germany remained untreated despite organ involvement (1). Due to the reduced QoL caused by psychosocial impairment in FD, such as gastrointestinal problems, pain, and depression (2,3), patients concluded that psychological support would be of benefit, but some patients received no support after their diagnosis. Additional challenges raised by the patient cohort included establishing a routine once therapy was initiated, and difficulties with self-cannulation and storing equipment at home for home infusions.

## References

1. Lenders M, Hennermann JB, Kurschat C, Rolfs A, Canaan-Kühl S, Sommer C, et al. Multicenter Female Fabry Study (MFFS) - clinical survey on current treatment of females with Fabry disease. *Orphanet J Rare Dis*. 2016;11(1):88.
2. Müller MJ. Neuropsychiatric and psychosocial aspects of Fabry disease. In: Mehta A, Beck M, Sunder-Plassmann G, editors. Fabry Disease: Perspectives from 5 Years of FOS. Oxford: Oxford PharmaGenesis. Copyright © 2006, Oxford PharmaGenesis™. 2006.
3. Deegan PB, Bähner F, Barba M, Hughes DA, Beck M. Fabry disease in females: clinical characteristics and effects of enzyme replacement therapy. In: Mehta A, Beck M, Sunder-Plassmann G, editors. Fabry Disease: Perspectives from 5 Years of FOS. Oxford: Oxford PharmaGenesis. Copyright © 2006, Oxford PharmaGenesis™. 2006.
